# Clinical management and outcome of patients with advanced NSCLC carrying EGFR mutations in Spain

**DOI:** 10.1186/s12885-018-4004-7

**Published:** 2018-01-30

**Authors:** Edurne Arriola, Ramón García Gómez, Pilar Diz, Margarita Majem, Maite Martínez Aguillo, Javier Valdivia, Alfredo Paredes, José Miguel Sánchez-Torres, Sergio Peralta Muñoz, Isidoro Barneto, Vanesa Gutierrez, Jesús Manuel Andrade Santiago, Francisco Aparisi, Dolores Isla, Santiago Ponce, David Vicente Baz, Angel Artal, Mariluz Amador, Mariano Provencio

**Affiliations:** 1 Medical Oncology Department, Hospital del Mar, Passeig Marítim, 25-29, 08018 Barcelona, Spain; 2 Hospital General Universitario Gregorio Marañón, Madrid, Spain; 3 Hospital Universitario de León, León, Spain; 4 Hospital de la Santa Creu i Sant Pau, Barcelona, Spain; 5 Complejo Hospitalario de Navarra, Pamplona, Spain; 6 Hospital Universitario Virgen de las Nieves, Granada, Spain; 7 Hospital Universitario Donostia, San Sebastián, Spain; 8 Hospital Universitario de La Princesa, Madrid, Spain; 9 Hospital Universitari Sant Joan de Reus, Reus, Tarragona Spain; 10 Hospital Universitario Reina Sofía, Córdoba, Spain; 11 Hospital Regional Universitario Carlos Haya, Málaga, Spain; 12 Hospital Virgen de la Salud, Toledo, Spain; 13 Hospital Virgen de los Lirios, Alcoy, Alicante Spain; 14 Hospital Clínico Universitario Lozano Blesa, Zaragoza, Spain; 15 Hospital Universitario 12 de Octubre, Madrid, Spain; 16 Hospital Universitario Virgen Macarena, Sevilla, Spain; 17 Hospital Universitario Miguel Servet, Zaragoza, Spain; 18 AstraZeneca, Madrid, Spain; 19 Hospital Universitario Puerta de Hierro Majadahonda, Madrid, Spain

**Keywords:** Clinical management, Chemotherapy, Epidermal growth factor receptor (EGFR) gene mutation, EGFR tyrosine kinase inhibitors (TKIs), Non-small-cell lung cancer (NSCLC)

## Abstract

**Background:**

Although the benefit of first-line epidermal growth factor receptor (EGFR) tyrosine-kinase inhibitors (TKIs) over chemotherapy has been demonstrated in several clinical trials, data from clinical practice is lacking and the optimal EGFR TKI to be used remains unclear. This study aims to assess the real-life diagnostic and clinical management and outcome of patients with advanced non-small-cell lung cancer (NSCLC) carrying *EGFR* mutations in Spain.

**Methods:**

All consecutive patients recently diagnosed with advanced or metastatic NSCLC from April 2010 to December 2011 in 18 Spanish hospitals and carrying *EGFR* mutations were retrospectively evaluated.

**Results:**

Between March and November 2013, a total of 187 patients were enrolled (98.3% Caucasian, 61.9% female, 54.9% never-smokers, 89.0% adenocarcinoma). Mutation testing was mainly performed on biopsy tumour tissue specimens (69.0%) using a qPCR-based test (90%) (47.0% Therascreen *EGFR* PCR Kit). Common sensitising mutations were detected in 79.8% of patients: 57.1% had exon 19 deletions and 22.6% exon 21 L858R point mutations. The vast majority of patients received first-line therapy (*n* = 168; 92.8%). EGFR TKIs were the most commonly used first-line treatment (81.5%), while chemotherapy was more frequently administered as a second- and third-line option (51.9% and 56.0%, respectively). Of 141 patients who experienced disease progression, 79 (56.0%) received second-line treatment. After disease progression on first-line TKIs (*n* = 112), 33.9% received chemotherapy, 8.9% chemotherapy and a TKI, and 9.8% continued TKI therapy. Most patients received first-line gefitinib (83.0%), while erlotinib was more frequently used in the second-line setting (83.0%). Progression-free survival (PFS) and overall survival (OS) in patients harbouring common mutations were 11.1 months and 20.1 months respectively (exon 19 deletions: 12.4 and 21.4 months; L858R: 8.3 and 14.5 months), and 3.9 months and 11.1 months respectively for those with rare mutations.

**Conclusion:**

EGFR TKIs (gefitinib and erlotinib) are used as the preferred first-line treatment while chemotherapy is more frequently administered as a second- and third-line option in routine clinical practice in Spain. In addition, efficacy data obtained in the real-life setting seem to concur with data from EGFR TKI phase III pivotal studies in NSCLC.

**Electronic supplementary material:**

The online version of this article (10.1186/s12885-018-4004-7) contains supplementary material, which is available to authorized users.

## Background

Non-small-cell lung cancer (NSCLC) accounts for more than 85% of lung cancer cases [1] with the majority of patients presenting with advanced disease at the time of diagnosis [2]. Standard first-line treatment for advanced disease has usually consisted of conventional cytotoxic chemotherapy, mostly platinum-based regimens, although it provides limited benefits with regard to survival [3, 4]. Advances in targeted and individualised treatment have led to the development of anti-epidermal growth factor receptor (EGFR) tyrosine kinase inhibitors (TKIs), such as first-generation TKIs (gefitinib, erlotinib) and second-line TKIs (afatinib), which irreversibly bind to the tyrosine kinase receptor. In addition, said advances have brought about the recently-available third-generation TKIs such as osimertinib, an oral, irreversible EGFR-TKI that is selective for both *EGFR* and *T790 M* resistance mutations and acts on the central nervous system. The development of these treatment strategies has markedly improved both clinical management and the outcome of patients with advanced NSCLC.

These targeted agents have shown a higher efficacy among patients harbouring specific activating mutations in exons 18–21 encoding the tyrosine kinase domain of the *EGFR* gene [5–10]. The most common *EGFR* activating mutations are exon 19 deletions (45%) and the L858R exon 21 point mutation (40–45%) [11, 12]. East Asians, females, never-smokers and patients with adenocarcinoma histology, who are associated with a higher incidence of *EGFR* activating mutations [13], have been shown to derive a greater clinical benefit from EGFR TKIs [5, 14, 15].

A large body of randomised clinical trials demonstrated the superior clinical effectiveness of EGFR TKIs compared with standard chemotherapy in patients with stage IIIB or IV NSCLC whose tumours tested positive for *EGFR* mutations [7–10, 13, 16, 17]. A large proportion of studies with these targeted agents were carried out in Asian patients from Japan, China and South Korea, where the incidence of *EGFR* mutations was high. However, some studies demonstrating the clinical benefit of TKIs over chemotherapy have been conducted in Caucasian populations (EURTAC [7], IFUM [18]), therefore confirming that the presence of *EGFR* mutations and not ethnicity is the most reliable factor predicting sensitivity to EGFR TKIs. All studies reported a superior benefit in overall response rate (ORR) and improvement [7–10, 13, 16, 17] in progression-free survival (PFS) for patients with *EGFR* mutation-positive NSCLC treated with TKIs compared with standard chemotherapy. None of the individual studies found a significant difference in overall survival (OS) between TKIs and chemotherapy, probably due to subsequent treatments and the high degree of crossover that may have confounded the effect of the initial first-line treatment.

The enhanced response to EGFR TKIs in patients harbouring activating mutations led to the recommendation of upfront *EGFR* mutation testing to guide therapeutic decision-making [19–22]. However, there is no consensus on the optimal detection method to identify *EGFR* mutations [23, 24] and the sources of tumour material (biopsy tumour tissue samples, cytology specimens or serum samples) have been a notable consideration in EGFR mutation detection.

Although the benefit of EGFR TKIs over chemotherapy has been clearly demonstrated in the first-line setting in several clinical trials, data from clinical practice is lacking and there are still some concerns regarding the optimal EGFR TKI to be used. Moreover, the most beneficial therapy (EGFR TKIs or chemotherapy) and the role of *EGFR* mutation status in second-line treatment and beyond still remain the subject of debate.

In addition to the lack of data available from routine clinical practice in patients with *EGFR*-mutated NSCLC, particularly in Caucasians, there is an unmet need for real-life data on treatment patterns and outcome at a national level in Spain. This study was conducted to assess the clinical management and outcome of patients with advanced NSCLC carrying *EGFR*-positive mutations in the real-world clinical setting in Spain.

## Methods

### Study design and patients

This was a multicentre, retrospective, observational study conducted in 18 hospitals throughout Spain. All adult patients (aged ≥18 years) recently diagnosed with histologically or cytologically confirmed advanced NSCLC from April 2010 to December 2011 and carrying *EGFR*-positive mutations were retrospectively evaluated.

The study was carried out in accordance with the Declaration of Helsinki and applicable regulatory requirements. Approval of the study protocol was obtained from the Hospital del Mar Clinical Research Ethics Committee (Barcelona, Spain). Written informed consent was obtained from all patients to retrospectively collect data from medical charts.

The primary endpoint of the study was to describe the diagnostic and clinical management patterns of patients with advanced or metastatic NSCLC carrying *EGFR*-positive mutations. For this purpose, *EGFR* mutation testing methods, source of tumour material, treatment setting and therapeutic strategies were analysed. Secondary endpoints included the clinical outcome (ORR, PFS and OS) of the overall population according to the line of therapy and treatment received (chemotherapy or EGFR TKIs [gefitinib or erlotinib]), the type of *EGFR* mutation (common or rare sensitising mutations, exon 19 deletion or L858R point mutation), and other relevant clinical characteristics (i.e. Eastern Cooperative Oncology Group [ECOG] performance status).

### Statistical analysis

A descriptive analysis was performed of diagnostic and clinical management variables collected from patient medical records. Quantitative variables were described using measures of central tendency and dispersion (mean, median, standard deviation [SD], minimum, maximum, first quartile and third quartile) and the results are expressed as mean ± SD or median (range). Qualitative variables are presented as absolute and relative frequencies. Efficacy analyses were conducted on the patients who had available data from at least one evaluation of response (8 weeks for EGFR TKI and 6 weeks for chemotherapy). Tumour response was assessed based on the unidimensional Response Evaluation Criteria In Solid Tumors (RECIST) version 1.1 [25] if the disease was measurable or by the investigator in those patients with a non-measurable disease according to local practice. ORR was calculated as the sum of patients achieving complete response and partial response as the best response achieved. PFS was assessed from the start of therapy for NSCLC until documented disease progression or death from any cause. Patients were censored at the date of last follow-up if still alive or without disease progression at the time of the analysis. OS was calculated as the time elapsed from the start of treatment to death. Patients were censored at the date of last follow-up if still alive at the time of the analysis. The probability of PFS and OS was estimated using the Kaplan-Meier method.

The statistical analysis was performed using the statistical package SAS version 9.02.

## Results

### Patient population

A total of 187 newly diagnosed advanced NSCLC patients (from April 2010 to December 2011) from 18 Spanish sites were retrospectively evaluated between March and November 2013. Six patients were excluded as they did not meet the inclusion criteria (the informed consent of two patients was not available and four patients were not diagnosed between April 2010 and December 2011). The demographic and clinical characteristics of the 181 evaluable patients are shown in Table 1. Briefly, 61.9% were female, 98.3% were Caucasian and 54.9% were never-smokers. The most frequent comorbidities were hypertension (48.1%), dyslipidaemia (21.0%), diabetes (14.4%), cardiovascular disease (11.6%), and chronic obstructive pulmonary disease (COPD) (10.5%). The most common histological type was adenocarcinoma (89.0%). ECOG performance status at diagnosis of advanced disease was 0 or 1 in 80.1% of patients. Most patients (87.8%) had stage IV disease at diagnosis. Metastases were mainly located in the lungs (45.7%), bone (42.9%) and pleura (28.6%).

**Table 1:** Patients´ demographic and clinical characteristics

Characteristic	Value (*n* = 181)
Median age (range), years	71.4 (62.2–79.0)
Gender, n (%)	
Male	69 (38.1)
Female	112 (61.9)
Race, n (%)	
Caucasian	178 (98.3)
Asian	3 (1.7)
Smoking history, n (%)	
Former smoker	53 (30.3)
Current smoker	26 (14.9)
Never smoker	96 (54.9)
ECOG PS at diagnosis of advanced disease, n (%)	
0	46 (27.7)
1	87 (52.4)
2	26 (15.7)
3	7 (8.1)
Tumor histology, n (%)	
Adenocarcinoma	161 (89.0)
Squamous cell carcinoma	9 (5.0)
Large cell carcinoma	5 (2.8)
Adenosquamous cell carcinoma	2 (1.1)
Carcinoma NOS	4 (2.2)
Clinical stage at diagnosis, n (%)	
IIIA	8 (4.4)
IIIB	7 (3.9)
IV	159 (87.8)
Other^a^	7 (4)
Median number of metastatic sites (range)	2.0 (1.0–3.0)
Metastases location, n (%)^b^	
Lung	80 (45.7)
Bone	75 (42.9)
Pleura	50 (28.6)
Lymph nodes	45 (25.7)
Pleural effusion	30 (17.1)
CNS	25 (14.3)
Liver	25 (14.3)

*CNS* Central nervous system, *ECOG PS* Eastern Cooperative Oncology Group Performance Status, *NOS* Not otherwise specified

^a^Other clinical stages included: IA (one patient), IIA (5 patients) and IIB (one patient), ^b^Metastatic locations presented in > 10% of patients

### EGFR mutation analysis

Mutation testing was mainly conducted in external laboratories (68.0%). The median time elapsed from the date the sample was sent to the laboratory until the results were obtained (turnaround time [TAT]) was 8.5 [7.0–12.0] days and 13.0 [5.0–20.0] days in external and internal laboratories, respectively. *EGFR* mutation testing was mainly performed using quantitative polymerase chain reaction (qPCR)-based tests (90.0%). The Therascreen^®^ *EGFR* RGQ PCR kit (QUIAGEN) was the most frequently used method for mutation testing (47.0%). *EGFR* mutation analysis was performed on biopsy tumour samples in 123 (68.7%) patients and on cytology specimens in 55 (30.7%) patients. Tumour tissue was primarily obtained from the primary tumour (72.9%). Tissue samples were mainly obtained through bronchoscopy (42.5%) or fine-needle aspiration (32.2%). The *EGFR* mutation testing methods and the source and type of tumour samples for mutation testing are shown in Table 2.

**Table 2:** Methods, source and type of tumor samples for EGFR mutation testing

Methods for EGFR mutation testing (*n* = 181)	*n* (%)
Therascreen EGFR Mutation Test kit (ARMS) (Qiagen)	85 (47.0)
RT-PCR (not specified)	26 (14.4)
Fluorescent PCR fragment length analysis	22 (12.2)
Direct sequencing	20 (11.0)
Cobas EGFR Mutation Test kit (Roche)	9 (5.0)
Allelic discrimination using fluorogenic probes	4 (2.2)
Digital PCR	2 (1.1)
Double PCR	1 (0.6)
Non specified	16 (8.8)
Sample characteristics	n (%)
Biopsy (*n* = 121)	123 (68.7)
Tumor tissue source	
Primary tumor	94 (77.7)
Metastatic sites	27 (22.3)
Cytology (*n* = 55)	55 (30.7)
Tumor tissue source	
Primary tumor	35 (63.6)
Metastatic sites	20 (36.4)
Biopsy and cytology type (*n* = 174)	
Bronchoscopy	74 (42.5)
FNA	56 (32.2)
Thoracocentesis	16 (9.2)
Surgery	14 (8.0)
Biopsy (unspecified)	8 (4.6)
Videothoracoscopy	5 (2.9)
Tru-cut	4 (2.3)
EBUS	1 (0.6)
Data not available	2 (1.1)

*EBUS* Endobronchial ultrasound, *FNA* Fine-needle aspiration

Among patients with available *EGFR* mutation type information (*n* = 168), sensitising mutations were detected in 157 (93.5%) patients. Of these, 134 (85.4%) patients harboured common sensitising mutations: 96 (61.1%) had exon 19 deletions and 38 (24.2%) exon 21 L858R point mutation. Other *EGFR* sensitising mutations found less frequently are described in Table 3.

**Table 3:** Common and rare sensitizing and not sensitizing mutations

Mutations (*n* = 168)^a^	*n* (%)
Sensitizing mutations	157 (93.4)
Common	134 (79.8)
Exon 19 (all mutations)	96 (57.1)
Exon 21 L858R	38 (22.6)
Rare	23 (13.7)
Exon 18 G719X	10 (5.6)
Exon 18 G719A	3 (1.8)
Exon 18 G719S	3 (1.8)
Exon 21 L861Q	7 (4.2)
Not sensitizing mutations	7 (4.2)
Exon 20 (all mutations)	7 (4.2)
Mutations of unknown significance	4 (2.4)
Exon 21 L858Q	1 (0.6)
Exon 21 E829Q	1 (0.6)
Exon 21 R836C	1 (0.6)
Exon 21 T854S	1 (0.6)

^a^Mutation type not available for 1 patient with exon 18 and 11 patients with exon 11 mutations

### Treatment

The vast majority of the patients had received first-line treatment after diagnosis of advanced NSCLC (92.8%). TKIs were used as first-line treatment in the majority of patients (81.5%), while chemotherapy-based regimens were more commonly administered as second- and third-line options (51.9% and 56.0%, respectively). First-line chemotherapy followed by maintenance EGFR TKIs was used in less than 5% of patients. Of the 168 patients who received first-line treatment, 79 (47.0%) underwent second-line treatment and 25 (14.9%) and 10 (6.0%) received third- and fourth-line treatment, respectively (Table 4).

**Table 4:** Treatment characteristics by line of treatment

Treatment	First-line	Second-line	Third-line	Fourth-line
	*n* (%)	*n* (%)	*n* (%)	*n* (%)
N	168	79	25	10
TKI	137 (81.5)	24 (30.4)	6 (24.0)	5 (50.0)
Gefitinib	114 (83.2)	4 (16.7)	1 (16.7)	0 (0.0)
Erlotinib	23 (16.8)	20 (83.3)	4 (66.7)	0 (0.0)
Afatinib	0 (0.0)	0 (0.0)	1 (16.7)	5 (100)
CT	31 (18.5)	43 (54.4)	14 (56.0)	4 (40.0)
Doublet	24 (77.4)	26 (60.5)	5 (35.7)	1 (25.0)
Monochemotherapy	4 (12.9)	16 (37.2)	8 (57.1)	2 (50.0)
Triplet	3 (9.7)	1 (2.3)	1 (7.1)	1 (25.0)
CT + maintenance TKI	8 (4.8)	1 (1.3)	0 (0.0)	0 (0.0)
CT + maintenance CT	2 (1.2)	1 (1.3)	0 (0.0)	0 (0.0)
Other	0 (0.0)	1 (1.3)	2 (8.0)	0 (0.0)

*CT* Chemotherapy, *TKI* Tyrosine kinase inhibitor

Of 141 patients who experienced disease progression on first-line treatment, 79 (56.0%) patients received second-line treatment. After disease progression on first-line EGFR TKIs (*n* = 112), 33.9% received chemotherapy, 8.9% chemotherapy and TKI, and 9.8% received further treatment with single agent TKI therapy. The majority of patients received first-line gefitinib treatment (83.2%), while erlotinib was the most frequent TKI used in the second-line setting (83.3%).

Of patients who received first-line chemotherapy (18.5%), doublet chemotherapy was used in 77.4% and 60.5% of patients as first and second-line treatment option, respectively.

In addition to pharmacological treatments, 36 (19.9%) patients underwent surgery (mainly palliative procedures involving minor surgery), and 71 (39.2%) received palliative radiotherapy.

### Efficacy

A total of 150/168 (89.3%) and 64/79 (81.0%) patients harbouring *EGFR* mutations were evaluable for efficacy analyses in first- and second-line setting, respectively.

At database lock, 120 (66.3%) patients had died, 29 (16.0%) patients were alive and had not experienced disease progression, 22 (12.2%) patients showed disease progression, and 10 (5.5%) patients were lost-to-follow-up. The median follow-up was 13.3 (0.4–38) months.

#### Clinical outcomes for patients according to treatment for advanced NSCLC

The ORR was 46.8% for patients treated with an EGFR TKI (gefitinib: 50.0%; erlotinib: 36.4%) and 22.2% for those receiving chemotherapy. Clinical efficacy in terms of response is detailed in Table 5. PFS was 9.9 (95% confidence interval [CI]: 8.3–11.5) months with first-line EGFR TKIs (gefitinib: 9.9 [95% CI: 8.3–11.7] months, erlotinib: 9.9 [95% CI: 4.8–15.0] months), 5.2 (95% CI: 3.8–7.1) months with standard chemotherapy and 7.6 (95% CI: 6.1–17.4) with chemotherapy followed by maintenance TKI therapy. Median OS was 16.7 (95% CI: 12.4–20.1) months and 23.7 (95% CI: 15.2–31.5) months with first-line gefitinib and erlotinib, respectively, 12.7 (95% CI: 9.3–21.0) months with chemotherapy and 16.6 (95% CI: 10.6–26.7) months for chemotherapy and maintenance TKIs (Table 5).

**Table 5:** Summary of efficacy by first-line treatment (TKI or chemotherapy) and TKI type (gefitinib or erlotinib) in the evaluable population

Endpoint	First-line treatment			TKI subtype	
	TKI	CT	CT + TKI maintenance	Gefitinib	Erlotinib
Evaluable	124	18	8	100	22
Response, n (%)					
CR	3 (2.2)	2 (11.1)	0 (0.0)	2 (2.0)	1 (4.5)
PR	55 (44.4)	2 (11.1)	2 (25.9)	48 (48.0)	7 (31.8)
SD	51 (41.1)	10 (55.6)	6 (75.0)	36 (36.0)	13 (59.1)
PD	15 (12.1)	4 (22.2)	0 (0.0)	14 (14.0)	1 (4.5)
ORR, n (%)	58 (46.8)	4 (22.2)	2 (25.0)	50 (50.0)	8 (36.4)
95% CI	37.8–55.9	6.4–47.6	3.2–65.1	39.8–60.2	17.2–59.3
DCR, n (%)	109 (87.9)	14 (77.8)	8 (100)	86 (86.0)	21 (95.5)
95% CI	80.8–93.1	52.4–93.6	63.1–100.0	77.6–92.1	77.2–99.9
Median PFS (95% CI), months	9.9 (8.3–11.5)	5.2 (3.8–7.1)	7.6 (6.1–17.4)	9.9 (8.3–11.7)	9.9 (4.8–15.0)
One-year PFS, n (%)	37.3	0.0	12.5	36.6	34.1
Median OS (95% CI), months	17.2 (13.5–21.4)	12.7 (9.3–21.0)	16.6 (10.6–26.7)	16.7 (12.4–20.1)	23.7 (15.2–31.5)
One-year OS, n (%)	61.7	50	37.5	59.8	66.6

*CI*: Confidence interval; *CR*: Complete response; *DCR*: Disease control rate; *ORR*: Overall response rate; *OS*: Overall survival; *PR*: Partial response; *PD*: Progressive disease; *PFS*: Progression-free survival; *SD*: Stable disease

#### Clinical outcomes for patients according to ECOG performance status

EGFR TKI treatment was more frequently used as first-line treatment in patients with poor ECOG PS (2 or 3) (100% in patients with PS 2 or 3 and 79% in those with PS 0 or 1). ORR was 46.8% (Disease control rate [DCR]: 87.2%) in patients with ECOG 0–1 and 47.6% (DCR: 95.2%) in those with ECOG 2–3. PFS was 9.9 (95% CI: 7.9–11.7) months for patients with ECOG 0 or 1 and 11.2 (95% CI: 9.5–19.7) months for those with worse ECOG. Finally, OS and one-year survival were 17.4 (95% CI: 13.4–25.5) months and 62.5% respectively in patients with ECOG 0–1 and 16.8 (95% CI: 10.7-not calculated) months and 54.9% in those with ECOG 2–3.

#### Clinical outcomes for patients according to EGFR mutation type

Of the 132 patients with *EGFR* sensitising mutations evaluable for efficacy, 112 (84.8%) had common sensitising mutations (exon 19 mutations and exon 21 L858R mutations) and 20 (15.2%) rare mutations (exon 18 G719X, G719A, G719S mutations and exon 21 L861Q mutation). Ninety-six (85.7%) and 16 (80.0%) patients with common and rare sensitising mutations received first-line TKIs, gefitinib being the most frequently used TKI in patients with common (81.3%) and rare (93.8%) mutations. Rare sensitising mutations present in tumours of patients treated with first-line TKIs were exon 18 G719A (2 [12.5%]), exon 18 G719S (2 [12.5%]), exon 18 G719X (7 [43.8%]) and exon 21 L861Q (5 [31.3%]) mutations.

**Fig. 1:**
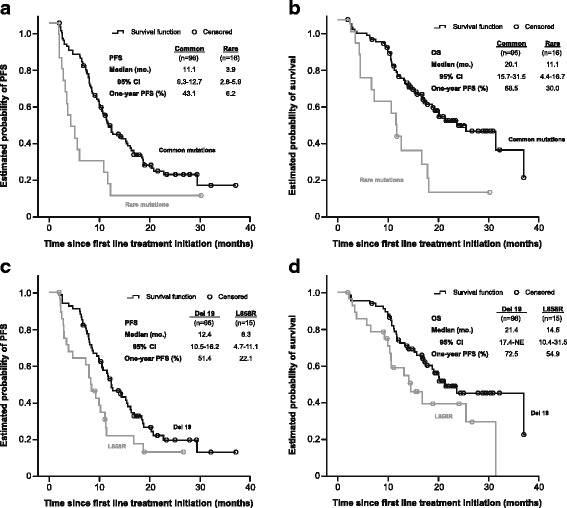
Kaplan-Meier curves for progression-free survival (**a**) and overall survival (**b**) for EGFR TKI-treated patients carrying common and rare EGFR sensitising mutations and Kaplan-Meier curves for progression-free survival (**c**) and overall survival (**d**) for patients treated with an EGFR TKI harbouring exon 19 deletions or L858R point mutations. *CI*: Confidence interval; *NE*: Not evaluable; *OS*: Overall survival; *PFS*: Progression-free survival

ORR was 53.1% in patients with common mutations (exon 19 deletions: 54.4%; L858R point mutations: 50.0%) and 12.5% in those carrying rare mutations (Table 6). PFS and OS in patients harbouring common mutations were 11.1 months and 20.1 months respectively, and 3.9 months and 11.1 months for those with rare mutations (Fig. 1a and b). PFS and OS in patients with exon 19 deletions was 12.4 (95% CI: 10.5–16.2) and 21.4 (95% CI: 17.4-not calculated) months, and 8.3 (95% CI: 4.7–11.1) and 14.5 (95% CI: 10.4–31.5) months respectively for those harbouring L858R (Fig. 1c and d).

EGFR TKI treatment resulted in an ORR of 53.1% in patients with common sensitising *EGFR* mutations who received TKIs while the ORR for those receiving chemotherapy was 18.8% (Table 6). The median PFS in patients carrying common sensitising mutations and treated with first-line TKIs was 11.1 (95% CI: 9.3–12.7) months while those receiving chemotherapy showed a PFS of 5.8 (95% CI: 4.2–7.6) months. The median OS was 20.1 (95% CI: 15.7–31.5) months with first-line EGFR TKIs and 12.1 (95% CI: 9.4-not achieved) months with chemotherapy.

**Table 6:** Summary of efficacy according to EGFR sensitizing mutation (common or rare) and common sensitizing EGFR mutation type (exon 19 deletion or L858R) and first-line treatment (EGFR TKI or chemotherapy) used in patients carrying common sensitizing EGFR mutations

	EGFR sensitizing mutation (*n* = 132) mutation (*n* = 131)		Common sensitizing EGFR mutation (*n* = 112)			
			Type of mutation		First-line treatment	
Endpoint	Common	Rare	Del 19	L858R	TKI	CT
Evaluable	112	20	82	30	96	16
Response, n (%)						
CR	3 (3.1)	0 (0.0)	2 (2.9)	1 (3.6)	3 (3.1)	1 (6.3)
PR	48 (50.0)	2 (12.5)	35 (51.5)	13 (46.4)	48 (50.0)	2 (12.5)
SD	38 (39.6)	8 (50.0)	27 (39.7)	11 (39.3)	38 (39.6)	11 (68.8)
PD	7 (7.3)	6 (37.5)	4 (5.9)	3 (10.7)	7 (7.3)	2 (12.5)
ORR, n (%)	51 (53.1)	2 (12.5)	37 (54.4)	14 (50.0)	51 (53.1)	3 (18.8)
95% CI	42.7–63.4	1.6–38.4	41.9–66.5	30.6–69.4	42.7–63.4	4.0–45.6
DCR, n (%)	89 (92.7)	10 (62.5)	64 (94.1)	25 (89.3)	89 (92.7)	14 (87.5)
95% CI	85.6–97.0	35.4–84.8	85.6–98.4	71.8–97.7	85.6–97.0	61.7–98.4

*CI* Confidence interval, *CR* Complete response, *DCR* Disease control rate, *ORR* Overall response rate, *PR* Partial response, *PD* Progressive disease, *SD* Stable disease

## Discussion

The present study examined the patterns of diagnostic and clinical management of patients with NSCLC carrying *EGFR*-positive mutations in routine clinical practice in Spain. As expected for patients carrying *EGFR* mutations and in line with previous reports in Caucasians [18], we found a population with a high proportion of females, never-smoker patients, and adenocarcinoma histology. Additionally, consistent with previous data in Caucasian patients, the majority of *EGFR* activating mutations were exon 19 deletions (57.1%) and exon 21 L858R point mutations (22.6%) [12].

As seen in our study, a variety of methodologies are used for *EGFR* mutation detection, as there is currently no consensus on the optimal method or source used for testing. The heterogeneity in the detection method has a potential relevance due to the differences in sensitivity between methods. Direct sequencing of DNA has traditionally been the “gold standard” for *EGFR* mutation testing, though this method is mainly limited by its moderate sensitivity and a long TAT [26, 27]. Indeed, only a small proportion of tumour samples (11.0%) were analysed using direct sequencing in daily-care patients analysed. *EGFR* mutation testing was primarily performed using RT-PCR-based tests (80.0%), the Therascreen^®^ *EGFR* Mutation Test kit (ARMS) being the most commonly applied mutation testing assay, which has demonstrated improved sensitivity and TAT [28]. Paraffin-embedded tumour tissue specimens have conventionally been the main source of tumour material for *EGFR* mutation testing in lung cancer and they currently still account for the majority of diagnostic samples in the clinical practice. Cytology specimens have been proven to be an adequate alternative source for mutation testing when tissue samples are not available or have a low content of tumour DNA [29, 30] and their use has increased over recent years. In our study, tumour tissue from bronchial biopsy was the most frequently used source of tumour material for *EGFR* mutation analysis (70.0%) while cytology specimens were used in about one third of patients at the time when the study was carried out.

The median TAT of 9 days seen in our study for sample analysis performed externally demonstrates a well-structured set up for this analysis in Spain. This allows physicians to have information available for treatment decision-making within an adequate time period after diagnosis, even in centres without local facilities to perform the analysis.

With regard to clinical management, one of the main findings of our study is the high proportion of patients who received EGFR TKIs in the first-line setting (82.0%), even though some of the current targeted agents were not available for patients harbouring EGFR mutations (e.g. erlotinib and afatinib) or had recently been approved at the time when the patients were diagnosed (e.g., gefitinib was marketed in March 2010). As expected, the most frequently used first-line TKI was gefitinib (83.2%). Therefore, EGFR TKI treatment may have been introduced early in the therapeutic armamentarium for advanced NSCLC in Spain. These findings therefore suggest that *EGFR* mutation testing may have been adopted early as a routine procedure to guide therapeutic decision-making in clinical practice in Spain even before it was widely adopted and recommended by major oncology groups, including the Spanish Society of Medical Oncology (SEOM) and the Spanish Society of Pathology (SEAP) [19–21, 31].

While TKIs were used as first-line treatment in the majority of patients, chemotherapy-based regimens were the preferred second-line option in our series. Only a small proportion of patients continued EGFR TKI therapy after disease progression, with a similar number of patients receiving single-agent TKI and TKIs plus chemotherapy. Acquired resistance to EGFR TKIs is the main limitation to a long-lasting benefit of these targeted agents in patients with *EGFR* mutation-positive NSCLC [32], with the *EGFR* T790 M mutation being responsible for resistance in up to 60% of cases [33]. However, withdrawal of an EGFR TKI at the onset of resistance may lead to rapid tumour growth [34, 35]. The potential benefit of continuing EGFR TKI treatment beyond disease progression has been addressed in several studies in the last years [36–38]. The single-arm phase II study ASPIRATION supports the feasibility of continuation of single-agent erlotinib beyond disease progression in patients with *EGFR* mutation-positive NSCLC [37]. However, further research based on randomised studies is needed before firm conclusions can be drawn. The phase III IMPRESS study showed that continuation of gefitinib in combination with platinum-based doublet chemotherapy after disease progression on first-line gefitinib did not prolong PFS compared with chemotherapy alone in patients with NSCLC carrying *EGFR* mutations [38]. The third-generation EGFR inhibitors, which can selectively target both sensitising mutations and the T790 M mutation, have demonstrated the benefit of continuing EGFR TKI treatment beyond progression for patients with T790 M mutation-positive NSCLC [39]. The recent approval of the third-generation EGFR TKI osimertinib may change the treatment paradigm after disease progression on EGFR TKI treatment in patients with T790 M mutation-positive NSCLC for which no other resistance mechanisms are identified.

Regarding the efficacy data, we found that tumour response and survival seem to be similar between gefitinib and erlotinib in real-life patients. However, the differences in the proportion of patients receiving first-line gefitinib and erlotinib in our series and the lack of matched comparisons make it difficult to obtain reliable data from our descriptive analysis. As a descriptive comparison only, considering the obvious limitations, the PFS achieved in this study with gefitinib (9.9 months) was within the range reported in the clinical trials carried out with gefitinib in Asian patients with advanced NSCLC [16, 40, 41] and the PFS data reported with gefitinib in a European population of Caucasian patients with advanced NSCLC harbouring *EGFR* mutations [18]. Similarly, erlotinib resulted in a comparable PFS to that reported in clinical trials with this targeted agent in Caucasians [7].

As expected, efficacy figures seem to be superior for patients harbouring common sensitising mutations in relation to those with rare mutations. Of note, only 12.5% of the patients carrying rare sensitising mutations (G719X) responded to EGFR-TKIs. Such a low response rate raises the question of whether these mutations should be considered “sensitizing” at all when it comes to EGFR first-generation TKIs.

Furthermore, PFS and OS appear to be longer for EGFR TKI-treated patients carrying exon 19 deletions compared with those with L858R point mutations. These findings are in line with previous clinical trials where numerical but non-significant differences in PFS were shown between patients treated with gefitinib harbouring exon 19 deletions and those with the L858R point mutation (11.5 months vs. 10.8 months, *p* = 0.90 in the NEJ002; Hazard ratio [HR] = 1.13, 95% CI = 0.63–2.03, *p* = 0.68 in the WJTOG3405) [5, 7, 16]. Similarly, a beneficial effect in favour of patients receiving the recently introduced TKI afatinib and carrying the exon 19 deletions was reported [8, 9].

In addition to the obvious limitations, arising from the retrospective nature of the study, the authors acknowledge that one of the main limitations of this study is the incorporation of the second-generation EGFR TKI afatinib for *EGFR*-mutated NSCLC in the last years which may have changed the prescription patterns regarding the type of EGFR TKI used. Despite these limitations, our findings still offer a valid global picture regarding the management of *EGFR*-mutant NSCLC patients who typically receive EGFR TKIs as their initial therapy. In addition, this study might offer a welcome addition to the limited “real-world” data on treatment patterns and clinical outcome of patients carrying *EGFR*-positive mutations in clinical practice, particularly in Caucasians. Our national data collection therefore provides an interesting overview of real-life clinical practice for the management of *EGFR*-mutated NSCLC in Spain.

## Conclusions

To our knowledge, this is the first study to have focused on the clinical management and outcome of real-life patients with advanced *EGFR*-mutated NSCLC in Spain. Our data show that EGFR TKIs were used as the preferred first-line treatment while chemotherapy was more frequently administered as a second- and third-line option. In addition, efficacy data, in terms of PFS and OS, obtained from our national real-world data collection, seem consistent with data from EGFR TKI phase III pivotal studies in NSCLC.

## Additional file

Additional file 1: Datasets supporting the study findings. (XLSX 269 kb)

## Abbreviations

CI: Confidence interval
CR: Complete response
DCR: Disease control rate
ECOG: Eastern Cooperative Oncology Group
EGFR: Epidermal growth factor receptor
NSCLC: Non-small-cell lung cancer
ORR: Overall response rate
OS: Overall survival
PCR: Polymerase chain reaction
PD: Progressive disease
PFS: Progression-free survival
PR: Partial response
RECIST: Response Evaluation Criteria In Solid Tumors
SD: Stable disease
TAT: Turnaround time
TKI: Tyrosine-kinase inhibitors
